# Smartphone App to Self-Monitor Nausea During Pediatric Chemotherapy Treatment: User-Centered Design Process

**DOI:** 10.2196/18564

**Published:** 2020-07-20

**Authors:** Astrid Eliasen, Mikkel Kramme Abildtoft, Niels Steen Krogh, Catherine Rechnitzer, Jesper Sune Brok, René Mathiasen, Kjeld Schmiegelow, Kim Peder Dalhoff

**Affiliations:** 1 Department of Clinical Pharmacology Bispebjerg and Frederiksberg University Hospital Copenhagen Denmark; 2 Department of Paediatrics and Adolescent Medicine University Hospital Rigshospitalet Copenhagen Denmark; 3 Institute of Clinical Medicine Faculty of Medicine University of Copenhagen Copenhagen Denmark; 4 ZiteLab ApS Copenhagen Denmark

**Keywords:** mobile applications, patient-reported outcome measures, patient compliance, neoplasms, antiemetics, nausea, vomiting, cancer, children, app

## Abstract

**Background:**

Nausea and vomiting are common and distressing side effects for children receiving chemotherapy. Limited evidence is available to guide antiemetic recommendations; therefore, prospective and reliable evaluation of antiemetic efficacy is needed. Smartphone apps can be used to effortlessly and precisely collect patient-reported outcomes in real time.

**Objective:**

Our objective was to develop a smartphone app to monitor nausea and vomiting episodes in pediatric cancer patients aged 0 to 18 years and to test its usability and adherence to its use.

**Methods:**

We used a user-centered design process and the evolutionary prototype model to develop and evaluate the app. Multidisciplinary group discussions and several rounds of patient feedback and modification were conducted. We translated the validated Pediatric Nausea Assessment Tool to assess nausea severity in children aged 4 to 18 years. The child’s own term for nausea was interactively incorporated in the nausea severity question, with response options expressed as 4 illustrative faces. Parent-reported outcomes were used for children aged 0 to 3 years. Reminders were sent using push notifications in order to ensure high response rates. Children aged 0 to 18 years who were undergoing chemotherapy were recruited from the Department of Pediatric Oncology at Copenhagen University Hospital Rigshospitalet to evaluate the app.

**Results:**

The app’s most important function was to record nausea severity in children. After assistance from a researcher, children aged 4 to 18 years were able to report their symptoms in the app, and parents were able to report symptoms for their children aged 0 to 3 years. Children (n=20, aged 2.0-17.5 years) and their parents evaluated the app prospectively during a collective total of 60 chemotherapy cycles. They expressed that the app was user-friendly, intuitive, and that the time spent on data entry was fair. The response rates were on average 92%, 93%, and 80% for the day before, the first day of, and the next 3 days after chemotherapy, respectively. Researchers and clinicians were able to obtain an overview of the patient’s chemotherapy dates and responses through a secure and encrypted web-based administrative portal. Data could be downloaded for further analysis.

**Conclusions:**

The user-friendly app could be used to facilitate future pediatric antiemetic trials and to refine antiemetic treatment during chemotherapy.

## Introduction

### Background

Chemotherapy-induced nausea and vomiting are among the most common and burdensome side effects that are experienced by children who are undergoing cancer treatment [1,2]. The symptoms are often accompanied by profound physical and psychological consequences, including dehydration, electrolyte imbalance, weight loss, and disruption of normal childhood activities [1,2]. The prevention of chemotherapy-induced nausea and vomiting has been improved over the years with novel antiemetic drug combinations [3-6], but approximately 50% of pediatric cancer patients still suffer from chemotherapy-induced nausea and vomiting [7-9].

Correct assessment is an essential first step to manage nausea experienced by children with cancer, but it is difficult to assess subjective symptoms such as nausea in children. Clinicians and children express and interpret nausea differently, which makes it a challenge to score the symptoms with high validity and reliability; therefore, antiemetic efficacy in pediatric trials is often solely based on vomiting (an objective outcome), while nausea (a subjective outcome) is rarely included as a clinical outcome [7,8,10]. Nausea is an important outcome measure because it occurs more often than vomiting and is one of the most distressing side effects for children with cancer [2]. Clinical trials should, therefore, assess antiemetic efficacy in children receiving chemotherapy using both nausea and vomiting as clinical outcomes.

The side effects of chemotherapy are often underdocumented by clinicians during prospective clinical trials [11-13]. Patient-reported outcomes can give more valid and reliable information on the side effects of chemotherapy [14]. Patient-reported outcomes are defined as symptoms that are directly reported by the patient, without interpretation by a clinician or anyone else and are used as clinical trial outcomes [14]. One barrier to accurately reporting the side effects experienced by patients as patient-reported outcomes is that the accuracy can be affected by the patient’s memory [14]. It is extremely important to record patient-reported outcomes at assigned times during pediatric clinical trials as recall bias may be more pronounced among children than among adults [15]. Also, pediatric cancer patients must often commute frequently between hospital and home, and some children live alternately with different parents or caregivers; in these situations, paper diaries may get lost or forgotten.

### Prior Work

Electronic diaries can collect patient-reported outcomes with more precision and effectiveness than paper diaries [16], and the increased number of smartphone users allows an opportunity to develop smartphone apps that collect real time patient-reported outcomes [17,18]. Even though pediatric patient-reported outcomes are advocated, parent-reported outcomes may be needed when the child is too young to complete a patient-reported outcome questionnaire [19].

There are several smartphone apps for adult patients with cancer that can monitor real time patient-reported outcomes and that are used in clinical trials and to support patients as they manage the side effects of chemotherapy [17,20,21]. In contrast, only a few smartphone apps have been developed to monitor patient-reported outcomes in children and adolescents with cancer [22]. Wang et al [23] developed a smartphone app where patients aged 8 years or older were able to record several symptoms such as pain, fatigue, depression, and anxiety. A user-centered design process has also been applied in the development of smartphone apps to assess and manage pain in children and adolescents [24,25]; Stinson et al [24] developed a game-based smartphone app to assess pain in adolescents with cancer [24]. The game-based nature and built-in reward system was appealing to users and promoted high adherence rates with a mean of 81% adolescents completing 100% of the tasks within a 2-week period [24].

### Study Goals

In this study, we aimed to develop and evaluate a smartphone app to monitor nausea severity in children from 0 to 18 years of age undergoing chemotherapy. We used a user-centered design process where we engaged children who were undergoing chemotherapy and their parents during the design, development, and evaluation of the app. The evaluation process included an investigation of the usability of the app and of adherence to the app’s use.

## Methods

### Overview

This study was conducted in Copenhagen, Denmark and was part of a prospective observational study. The study was approved by the Danish Data Protection Agency (RH-2016-219, I-Suite: 04804) and the Danish National Research Ethics Committee (H-18020267). The app was approved by the regulatory authorities in *Center for It, Medico og Telefoni* in the Capital Region of Denmark.

**Figure 1:**
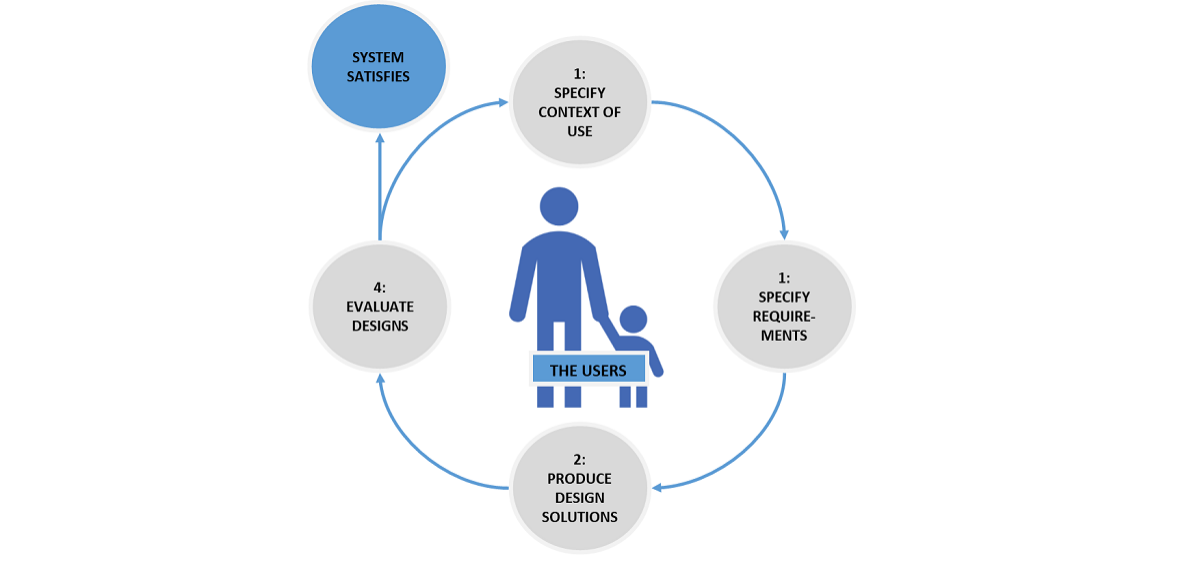
The app was developed and evaluated with the user-centered design process (adapted from ISO 9241-210:2019).

**Figure 2:**
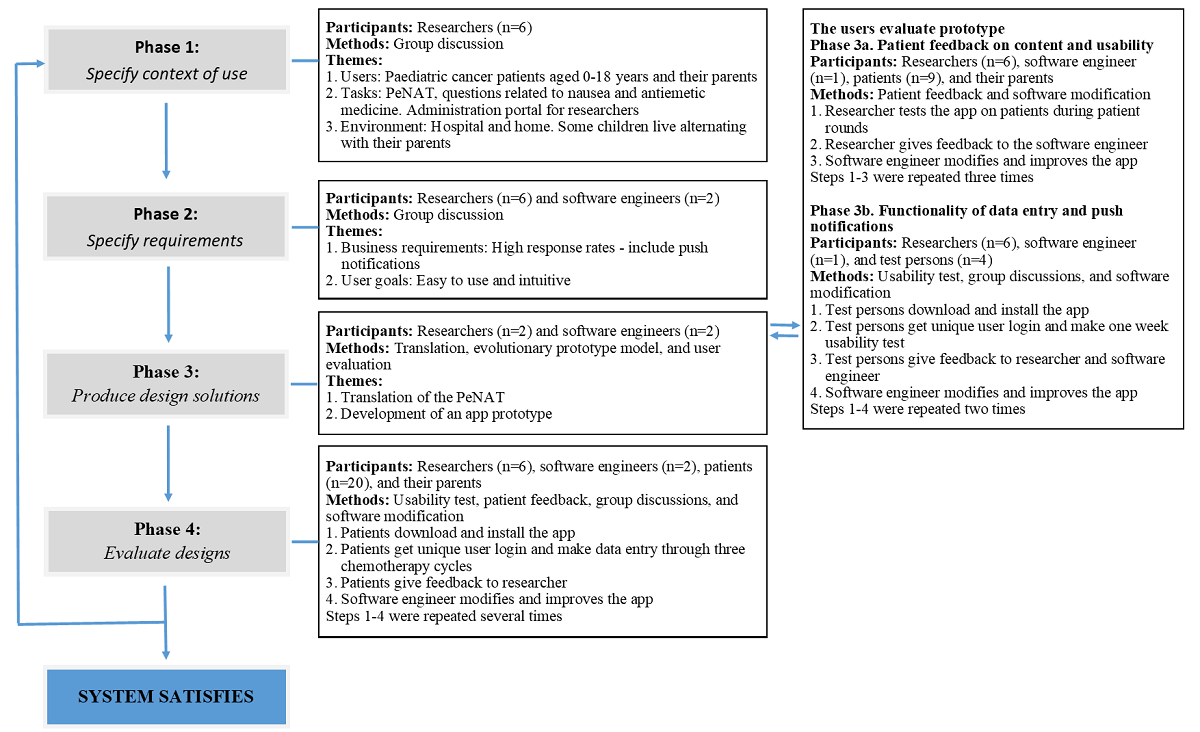
Workflow of the development and evaluation process according to the user-centered design process and the evolutionary prototype model.

We used a user-centered design process and the evolutionary prototype model to develop and evaluate the app [26,27], and we involved pediatric cancer patients and their parents throughout the process (Figure 1 and Figure 2). Medical researchers and software engineers worked synergistically in the development of the app. Pediatric oncologists were consulted throughout the process because they have an explicit understanding of the users (pediatric cancer patients). The app was developed in 4 phases: (1) The context of use was specified (ie, the users, the tasks that the users would perform, and the environment in which the users would use the app were characterized), (2) requirements were specified (ie, requirements and user goals that must be met for the app to be successful were identified), (3) design solutions were produced (ie, an app prototype was developed), and (4) the app was evaluated (ie, for data completeness and to further refine the app).

### Specify Context of Use (Phase 1)

Researchers including pediatric oncologists (n=4), a clinical pharmacologist (n=1), and a medical researcher (n=1) participated in the first discussions. The advantages of developing an app and its content, as well as strategies to obtain complete data sets were discussed. The discussion was based on previous experience with pediatric antiemetic trials and a literature review that was used to identify the best validated tool for pediatric patient-reported nausea severity.

### Specify Requirements (Phase 2)

The requirements were discussed with 2 software engineers from ZiteLab in Copenhagen, Denmark [28]. A requirement was that the app should include a customized notification system to ensure high response rates. We aimed for a user-friendly and intuitive app, and the usability was refined during several rounds of patient feedback and modification (phases 3a and 3b).

### Produce Design Solutions (Phase 3)

#### Overall Structure

The app was developed with hybrid web-based technology to allow a single code base to be deployed for both iPhone and Android operating systems. Feedback was received from the users (phases 3a and 3b), and subsequent prototypes were produced, each with additional functionality or improvements until a fully functional app was developed [27].

**Figure 3:**
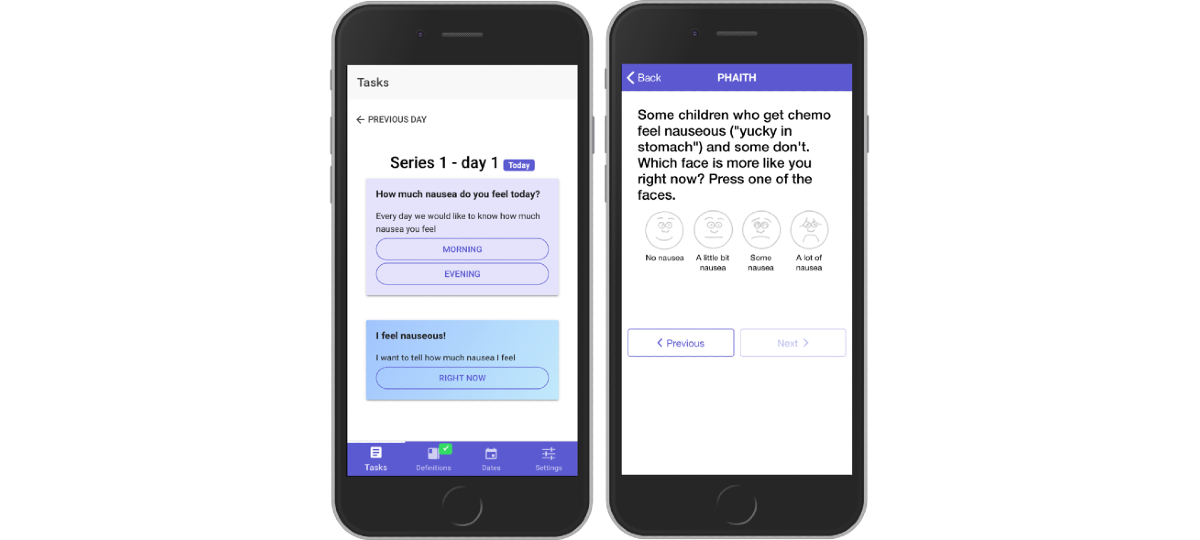
The app with overview of the day’s task (left) and the patient’s expression of nausea severity (right).

The users were able to get an overview of the day’s tasks when the app was opened (Figure 3). Each morning, the user reported nausea severity. Each evening, the user reported nausea severity, retching and vomiting episodes, and antiemetic medications. In addition, users could report nausea severity at any time they (or in the case of parents for children from 0 to 3 years of age, their child) felt nauseated.

#### Data Entry on Nausea Severity

We translated the Pediatric Nausea Assessment Tool (PeNAT), a valid and reliable tool to assess nausea severity among children who are pediatric oncology patients (from 4 to 18 years of age) [29], from English to Danish. A questionnaire in the PeNAT ensured that the child understood the concept of nausea, and it focused the child’s attention to their feeling of nausea. Four faces in the PeNAT corresponded to the nausea severity encountered by the child—no nausea, mild nausea, moderate nausea, and severe nausea (Figure 3). Children older than 8 years of age were presented with the 4 faces simultaneously, while children aged 8 years or younger were presented with 2 faces simultaneously, and the pair presented depended on whether the child said they felt no nausea or some nausea.

We translated the PeNAT according to The International Medical Interpreters Association Guide on Medical Translation [30]. The developers of the PeNAT were contacted for an explanation of the concepts before the translation process. A pediatric oncologist (CR) and a medical researcher (AE), both with substantial knowledge of the subject terminology, independently translated the tool from English to Danish. The translations were compared and merged into a single final translation, and if necessary, a third researcher (MKA) was consulted.

The app had a definition module where the child was able to define the term used for nausea as they understood it, in accordance with the PeNAT [29]. The child could then express nausea severity by tapping one of 4 faces that were presented horizontally and that represented increasing nausea severity from left to right; the child’s own terminology for nausea was incorporated in the question (Figure 3).

The app was also developed to address the need for parent-reported outcomes for children aged 0 to 3 years. For example, one question in the PeNAT is “Which face is more like you right now?”; in the parent-reported tool, this question is worded as “Which face is more like your child right now?”

Accordingly, 3 versions of the app were developed: one for children aged 0 to 3 years (parent-reported outcome), one for children aged 4 to 8 years (2 faces presented simultaneously), and one for children aged 9 to 18 years (4 faces presented simultaneously).

#### Data Entry on Questions Related to Nausea

The patients were asked about vomiting and retching episodes each day. These questions were answered with 4 single bullet options: no; yes, 1 to 2 times; yes, 3 to 5 times; or yes, 6 or more times. An episode was deﬁned as a vomit or retch that was separated from another vomit or retch by at least one minute.

#### Push Notifications

Push notifications were sent at 9 AM and 7 PM if the questionnaires had not been answered each morning and evening, respectively.

#### Web-Based Administration Portal

The web-based administrative portal was created as a secure and encrypted home page. Data were anonymized (with pseudonyms) and data storage and transmission followed general data protection regulations. The portal consisted of a front end (presentation layer for researchers) and a back end (data access layer for software engineers).

The researchers could create unique log-ins for patients including username, password, age group (0 to 3 years, 4 to 8 years, or 9 to 18 years), and scheduled chemotherapy dates. The dates could be changed by the researchers, and the users could forward changes to the researchers with a simple tap in the app, if the dates were incorrect. The researchers could get an overview of the patient’s scheduled chemotherapy dates and view the patient’s responses and response rates. The data could be downloaded for further analysis.

#### Patient Feedback on Content and Usability (Phase 3a)

The pilot assessment feedback came from children who were pediatric patients and their parents, all of whom were recruited at the Department of Pediatric Oncology, Copenhagen University Hospital Rigshospitalet, during regular patient rounds in November 2018 and December 2018. We used a qualitative usability testing approach with 3 cycles of patient feedback and modification. Children (n=9) and their parents participated (3 in each of 3 cycles). AE introduced the app to the test participants who were asked to comment on words, to interpret phrases in the questionnaire, and to suggest improvements to the interface. A software engineer (MKA) modified the app according to the feedback. The app prototype was modified until no further changes were suggested.

#### Functionality of Data Entry and Push Notifications (Phase 3b)

Clinical researchers (n=4) from the Department of Clinical Pharmacology, Bispebjerg and Frederiksberg University Hospital tested the functionalities of log-in, data entry, and push notifications during 2 test periods, each lasting one week. Test persons (n=4), 2 with an Android and 2 with an iPhone smartphone, downloaded the app and were given unique log-in information. The test person registered if they were able to log-in, if they received push notifications, and if they could enter data in the app. MKA and AE performed group discussions with these individuals after each test period and discussed the functionality of the app. MKA modified the app according to the feedback.

### Evaluate Designs (Phase 4)

Following the development of a fully functional app, we included patients to test usability of the app and adherence to its use. Patients (n=20) were recruited at the Department of Pediatric Oncology, Copenhagen University Hospital Rigshospitalet during March 2019 and April 2019. All parents provided written informed consent for their child to participate in the study. Eligible patients were children aged 0 to 18 years who would be receiving at least 3 cycles of chemotherapy. The child and at least one parent was required to be able to communicate fluently in Danish. At least one parent needed to have an Android or iPhone smartphone. Parents of children aged 0 to 3 years needed to be able to describe the nausea severity experienced by their child.

One or both parents downloaded and installed the app from Google Play or App Store and were given unique log-in information. It was emphasized that the data collected were to be used for research purposes, and that the parents should also inform their pediatric oncologist or nurse about their child’s symptoms. The questions in the app were delivered to each child by AE who also taught the parents to administer the app. The children (with assistance from their parents) were asked to record their own nausea severity in the app, and AE noted if they were able to naturally navigate through the app’s features. The parents could then assist the child to enter data about nausea severity for the day before and for the first 4 days after the start of chemotherapy during 3 chemotherapy cycles. The children and their parents were contacted in person before each cycle to promote adherence to use of the app. A semistructured interview was conducted at that time, and the participants were asked what they liked or disliked about the app, if the app was easy to use, and if the time spent on data entry was fair. AE provided continuous feedback to MKA who modified the app according to the feedback. This was continued until there were no further recommendations for changes in the app.

The web-based administrative portal was fully developed in this phase. AE tested if log-ins for new patients could be created and if the portal provided a user-friendly interface and overview of data completeness. MKA tested whether the back end received the entered data accurately and if data could be downloaded for further analysis.

We subsequently interviewed parents of patients aged 0 to 3 years in order to determine if the parents could grade the nausea severity experienced by their child.

A paired, two-tailed *t* test was used to determine if the response rates differed between morning and evening and if the response rates differed between chemotherapy cycles. *P*<.05 was deemed significant.

## Results

### Context of Use, Requirements, and Design Solutions (Phases 1, 2, and 3)

During phases 1 and 2, we specified in which context the app would be used, and we agreed on the overall requirements of the app (Figure 1 and Figure 2). Based on the functions and requirements that were described by the researchers, in phase 3, a beta-version smartphone app was created by software engineers from ZiteLab in Copenhagen, Denmark [28]. These functions and requirements were refined and modified according to feedback from children who were pediatric cancer patients, parents, and participants (phase 3a and phase 3b).

### Participant Characteristics

The demographic and disease characteristics of the children who were pediatric cancer patients (phases 3a and 4) and the demographic characteristics of clinical researchers (phase 3b) who participated in the user-centered testing are shown in Table 1.

**Table 1:** Baseline demographics and disease characteristics of participants who were included in the development and evaluation of the app.

Characteristics			Phase 3a (n=9)		Phase 3b (n=4)		Phase 4 (n=20)
Age in years^a^, mean (range)			7.2 (2.9-16.5)		32 (30-36)		7.4 (2.0-17.5)
**Gender, n (%)**							
	Female	4 (44)		4 (100)		8 (40)	
	Male	5 (56)		0 (0)		12 (60)	
**Primary diagnosis, n (%)**							
	Acute lymphoblastic leukemia	5 (56)		N/A^b^		9 (45)	
	Brain tumor	1 (11)		N/A		3 (15)	
	Hodgkin lymphoma	1 (11)		N/A		3 (15)	
	Kidney tumor	0 (0)		N/A		1 (5)	
	Non-Hodgkin lymphoma	1 (11)		N/A		1 (5)	
	Osteosarcoma	1 (11)		N/A		2 (10)	
	Rhabdomyosarcoma	0 (0)		N/A		1 (5)	
Duration of illness in days^a^, mean (range)			111 (7-251)		N/A		110 (1-299)

^a^At the time of study recruitment.

^b^N/A: not applicable.

### Patient Feedback on Content and Usability (Phase 3a)

The questions in the PeNAT were clear and easy to understand, and the patients and their parents had no comments relating to the meaning of words or phrases in the translation. The parents of one newly diagnosed patient (less than one week) reported that it would be demanding to enter data, which was in contrast with the feedback from parents of patients who had been through several rounds of chemotherapy, who expressed that they could easily overcome to enter data in the app.

One suggestion for the interface was to include emojis as pictorial expressions of the questions. Emojis were not included in the PeNAT part of the app to be true to the original PeNAT paper version.

Through interviews with parents, we found that the actual antiemetics that were given did not always correspond with the prescribed medications in the medical record, especially when the child was discharged from hospital. To capture the actual medication use, a medicine module was developed to let parents fill in administered antiemetics, numbers of administrations, and whether the patient used antiemetic rescue medications on any given day. The medicine module included an autofill function where the parents could copy answers from the previous day with a single tap and modify answers that had changed.

### Functionality of Data Entry and Push Notifications (Phase 3b)

During the first test period, problems with the log-in function required that participants restarted the app. All test participants received push notifications informing them to enter data in the app, and they were subsequently able to do so.

### Design Evaluation (Phase 4)

The app was evaluated prospectively in patients (n=20) during a total of 60 chemotherapy cycles.

#### Usability

Overall, the patients and their parents expressed that the app was user-friendly, intuitive, and that time spent on data entry was fair. Satisfaction with the app was illustrated with the following quotes: A male participant aged 12 years said, “It is a really cool app, and the questions are very relevant.” A parent of a 4-year old female said that, “she really likes to use the app, and she also uses the ad hoc function to tell how she is feeling.”

The semistructured interviews revealed suggestions for changes in the app. First, the log-in session was expanded from 2 hours to 3 months, so that the users did not need provide log-in information at every new task. Second, 15% (3/20) of patients were so nauseated that they could not overcome the nausea to answer the questions on that specific day; therefore, the parents requested a solution enabling them to go back and help the child answer questions from the previous day. This option was then included in the app allowing users the possibility to return to answer questions from within the previous 2 days. Third, the days with questions were individualized, so they continued for 5 days after the last day with chemotherapy in the cycle. This feature was added because 15% (3/20) of the children who received chemotherapy over several days felt nauseated longer than four days after the first chemotherapy administration.

Two adolescent patients suggested that the app should include a module with an overview of their answers. It is our intention to include this feature in a future version of the app.

#### Adherence to Use

**Figure 4:**
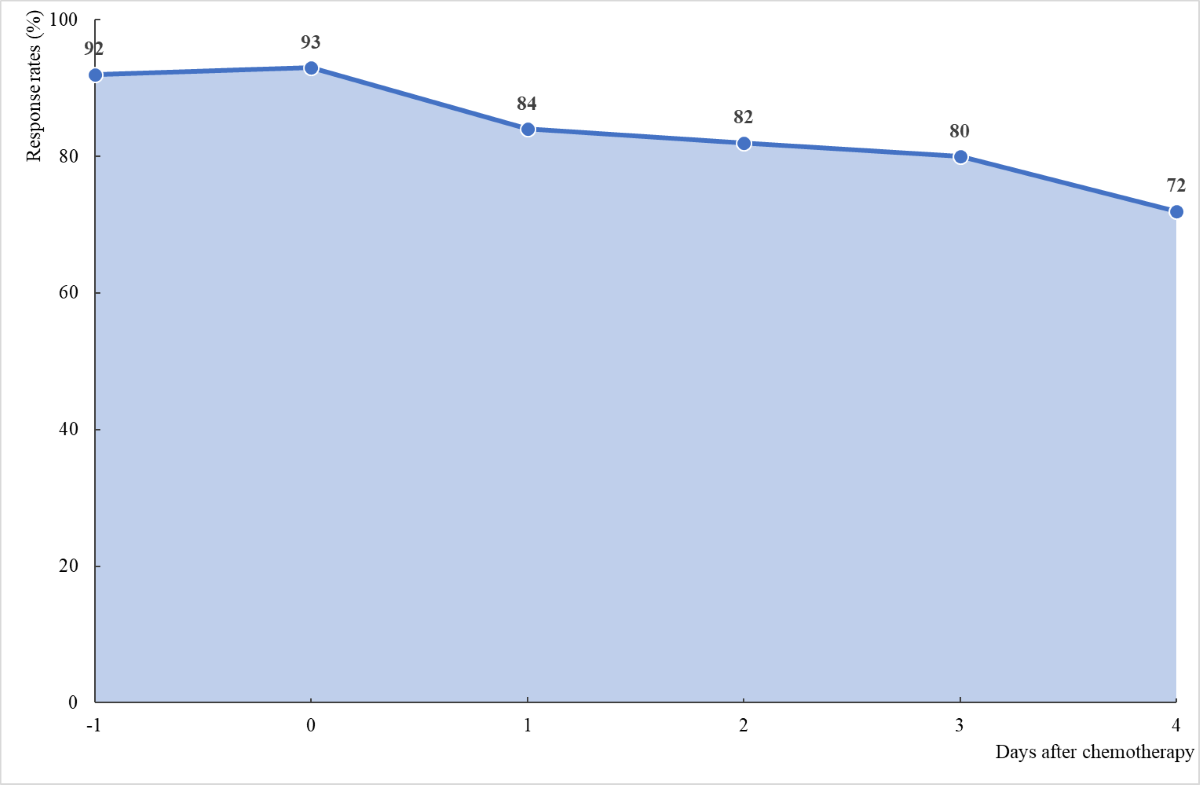
Data completeness for 20 patients using the app during 60 chemotherapy cycles.

All patients and their parents expressed that push notifications were a convenient and efficient method to ensure that they completed their tasks. The response rates were on average 92%, 93%, and 80% for the day before, the first day, and the next 3 days after chemotherapy, respectively (Figure 4). Individual response rates of 100%, 50%, or 0% were given each day if the patient answered both morning and evening, answered either morning or evening, or did not answer, respectively.

The personal contact between the researcher and the participants before each chemotherapy cycle promoted high response rates the day before and the day of chemotherapy administration. The next 3 days after chemotherapy, 15% (3/20) of the children did not respond, each with different reasons—one patient did not receive the push notifications, one patient had difficulties with the log-in function, and one patient had a limited internet connection at home. One adolescent (1/20, 5%) downloaded the app on her own smartphone but did not complete the questions due to severe nausea. Her parents then downloaded the app and assisted her.

There were no significant changes in response rates when response rates for cycles 1 to 3 were analyzed separately (*t*_5_=2.57, *P*=.45 for comparing response rates for cycle 1 with those of cycle 3). The response rates in the morning were slightly higher than those in the evening, but the difference was insignificant (*t*_5_=2.57, *P*=.11).

#### Additional Data on Usability for Patients Aged 0 to 3 Years

The interviews showed that most parents (21/22, 95%) of the children aged 0 to 3 years could explain how their child reacted when feeling nauseous, and they could use the app to express their child’s nausea severity. Only the parents of one child (1/22, 5%) did not know how their child reacted when feeling nauseous.

## Discussion

### Principal Results

We described the process of developing and evaluating a smartphone app to collect pediatric patient-reported outcomes of nausea severity and episodes of vomiting and retching. Patients were able to self-report in the app the severity of nausea that they experienced with assistance from their parents, and clinicians and researchers had a good historical overview of their symptoms in the web-based administrative portal. Multidisciplinary group discussions ensured that the app and the web-based administrative portal met both medical and technical demands. It was essential that the app had a high usability, so patients and their parents were involved early in the development to get insight into their preferences.

Generally, patients prefer mobile data collection over more traditional methods such as paper diaries, because it is easier and faster to record data into a mobile device than it is to record in a paper diary [31]. Some children thought it was interesting to answer questions in the app because they liked to press the faces reflecting nausea severity. They easily understood the interactive component because the child’s own term for nausea was incorporated into the questions. These features are not possible in a paper diary or a web-based survey.

### Usability of the Smartphone App

Nausea has a higher incidence than vomiting during administration of chemotherapy, but it is a challenge to recognize subjective symptoms such as nausea in children. Furthermore, children under 4 years of age are often excluded from studies that investigate the burden of chemotherapy-induced nausea and vomiting in children [32-34]. The target users of this app are children aged 0 to 18 years (and their parents) undergoing cancer treatment. Our subjective impression was that children aged 4 to 18 years were able to express their nausea severity in the app. One 17-year-old patient expressed (about nausea) that “it hurts in my stomach and I have no appetite,” whereas a 7-year-old patient expressed that nausea was “like an elevator moving up through the stomach and all the way to the throat.” Children aged 0 to 3 years are too young to complete a patient-reported outcome instrument, and the PeNAT is not validated for this age group; however, parents of patients aged 0 to 3 years expressed that they could easily grade the nausea severity experienced by their child. By including this age group, researchers were able to obtain data about parent-reported nausea severity and vomiting episodes for children aged 0 to 3 years.

### Patient Adherence With Paper-Based and Electronic Diaries

It was important that the patients adhered to the use of the app, including patients with severe nausea who likely had reduced energy to perform extra tasks during chemotherapy. In a paper version of the PeNAT, 17% of the patients did not use the questionnaire for three or more days out of seven days [32]. The high response rates in our study were attributed to the individual patient reminders. Also, data were captured in real time, and we could identify and respond to nonadherence early by following the patient responses in the web-based administrative portal. The reasons for nonadherence were mainly technical difficulty–based (mainly in the first version of the app) and not that the tasks were too demanding. The response rates did not decrease over three chemotherapy cycles; therefore, the app has the advantage that it could be used in antiemetic trials using a crossover design, where patients undergo two chemotherapy cycles.

Real time patient-reported outcomes reduce the risk of recall bias and improve data quality. After requests from patients and parents, patients were allowed to return to answer questions from within the previous 2 days. This allowed patients with severe symptoms on one day to respond the following day. The survey time was recorded in the administration system which permitted analysis of data accuracy and the ability to exclude data that was not registered in real time. This is in contrast to a paper-based approach, where the researchers are not aware of whether patients are back filling or completing the paper diary just prior to returning it to the researchers [24].

### Challenges in Using Apps to Collect Patient-Reported Outcomes

The digital revolution offers exciting new tools to support research, but along with these new opportunities come some challenges. We conducted a preliminary assessment of the suitability of apps in the target population, and every participant in this study had a smartphone. Other target populations may not have access to digital technology, and the target population should, therefore, be assessed to determine whether digital data collection is appropriate.

The development of an app is time consuming compared to the time required to develop a paper diary or a simple electronic web-based survey. Simple electronic diaries for use on smartphones can be created at online research platforms without extensive programming skills [35,36]. More advanced options, such as individual patient reminders and interactive components, require assistance from experienced software engineers. Also, data storage in these platforms may not adhere to general data protection regulations. The cost of developing an app depends on the complexity of the interface and functions, and additional costs must be considered for data storage and distribution in Google Play and App Store; however, electronic data collection reduces the time used for data handling because researchers do not need to manually record data from paper diaries [31]. This more precise and efficient data collection method could also outweigh the time and price spent on preparing the trial [16-18].

Technical support needs to be available to maintain and upgrade the app over time in clinical trials using apps for data collection. Technical problems mainly arose when the software was updated or when a new version of the app was launched. The major technical problems were that patients could not log-in to the app and that push notifications were not sent. Other barriers that could prevent efficient data entry are limited access to internet or if a patient signs out and forgets the username and password. These problems require that investigators of the clinical trial pay attention to both the function of the app and to feedback from patients throughout the clinical trial so that technical problems can be solved immediately. In our study, one researcher was signed into the app with a test user log-in to capture technical difficulties which were then directly forwarded to a software engineer. A researcher also directly transcribed patient-reported outcomes from the patients at any time they were not able to report patient-reported outcomes in the app.

The data entered by the patients will be used to determine patient-related risk factors of chemotherapy-induced nausea and vomiting, and the app will be used to collect pediatric patient-reported outcomes in upcoming antiemetic trials. The overview of the patient-reported outcomes can be used in the future by clinicians to better refine antiemetic treatment for forthcoming chemotherapy.

### Conclusions

The app and the web-based administrative portal demonstrated good usability for patients and researchers, and pediatric patient-reported outcomes of nausea severity were collected efficiently. The app can be used to facilitate future research regarding antiemetic efficacy in pediatric cancer patients and to better refine antiemetic treatment in upcoming chemotherapy cycles.

## Abbreviations

PeNAT: Pediatric Nausea Assessment Tool
